# MIDRC mRALE Mastermind Grand Challenge: AI to predict COVID severity on chest radiographs

**DOI:** 10.1117/1.JMI.12.2.024505

**Published:** 2025-04-18

**Authors:** Samuel G. Armato, Karen Drukker, Lubomir Hadjiiski, Carol C. Wu, Jayashree Kalpathy-Cramer, George Shih, Maryellen L. Giger, Natalie Baughan, Benjamin Bearce, Adam E. Flanders, Robyn L. Ball, Kyle J. Myers, Heather M. Whitney, the MIDRC Grand Challenge Working Group

**Affiliations:** a The University of Chicago, Department of Radiology, Chicago, Illinois, United States; b University of Michigan, Department of Radiology, Ann Arbor, Michigan, United States; c University of Texas MD Anderson Cancer Center, Department of Thoracic Imaging, Houston, Texas, United States; d University of Colorado Anschutz Medical Campus, Department of Ophthalmology, Aurora, Colorado, United States; e Weill Cornell Medicine, Department of Radiology, New York, New York, United States; f Thomas Jefferson University, Department of Radiology, Philadelphia, Pennsylvania, United States; g The Jackson Laboratory, Bar Harbor, Maine, United States; h Puente Solutions, Phoenix, Arizona, United States

**Keywords:** grand challenge, coronavirus disease-19, artificial intelligence

## Abstract

**Purpose:**

The Medical Imaging and Data Resource Center (MIDRC) mRALE Mastermind Grand Challenge fostered the development of artificial intelligence (AI) techniques for the automated assignment of mRALE (modified radiographic assessment of lung edema) scores to portable chest radiographs from patients known to have COVID-19.

**Approach:**

The challenge utilized 2079 training cases obtained from the publicly available MIDRC data commons, with validation and test cases sampled from not-yet-public MIDRC cases that were inaccessible to challenge participants. The reference standard mRALE scores for the challenge cases were established by a pool of 22 radiologist annotators. Using the MedICI challenge platform, participants submitted their trained algorithms encapsulated in Docker containers. Algorithms were evaluated by the challenge organizers on 814 test cases through two performance assessment metrics: quadratic-weighted kappa and prediction probability concordance.

**Results:**

Nine AI algorithms were submitted to the challenge for assessment against the test set cases. The algorithm that demonstrated the highest agreement with the reference standard had a quadratic-weighted kappa of 0.885 and a prediction probability concordance of 0.875. Substantial variability in mRALE scores assigned by the annotators and output by the AI algorithms was observed.

**Conclusions:**

The MIDRC mRALE Mastermind Grand Challenge revealed the potential of AI to assess COVID-19 severity from portable CXRs, demonstrating promising performance against the reference standard. The observed variability in mRALE scores highlights the challenges in standardizing severity assessment. These findings contribute to ongoing efforts to develop AI technologies for potential use in clinical practice and offer insights for the enhancement of COVID-19 severity assessment.

## 1 Introduction

The publicly available Medical Imaging and Data Resource Center (MIDRC) database, funded by the National Institute of Biomedical Imaging and Bioengineering (NIBIB), was initiated in 2020 in response to the coronavirus disease-19 (COVID-19) pandemic.^1^ The MIDRC database has become a valuable collection of medical images available to the medical imaging research community. The aim of MIDRC is “to foster machine learning innovation through data sharing for rapid and flexible collection, analysis, and dissemination of imaging and associated clinical data by providing researchers with unparalleled resources in the fight against COVID-19 and beyond”.^1^

One of the mandates for MIDRC was to organize grand challenges using the MIDRC database. Through these scientific grand challenges, organizers influence medical imaging research by designing a challenge that targets a relevant and timely topic so that participating research groups dedicate their skills and knowledge toward the designated task.^2^ Unlike most challenges that are constrained by existing datasets, MIDRC leverages its large database to first conceptualize and develop a grand challenge, maximizing the potential for community interest and clinical relevance. Cases (including medical images and associated data) submitted to MIDRC from contributing clinical sites undergo a multi-faceted curation process. A portion of each newly submitted cohort is sequestered for future use in the United States Food and Drug Administration (FDA) regulatory approval process, and the remaining cases in the cohort are scrutinized before being either reserved for a planned grand challenge or marked for immediate release to the public data commons. Reserved cases that meet the criteria for a planned grand challenge become part of the challenge’s validation or test set and are withheld from public release until completion of the challenge. Training sets for grand challenges may be assembled from cases already available to the public. One advantage of MIDRC challenges is the ability to customize training, validation, and test sets from curated cases, which provides an opportunity to explicitly account for potential bias from multiple sources.^3^^–^^6^ All images and associated metadata such as patient demographics and COVID test results were de-identified to safe harbor standards^7^ prior to ingestion to MIDRC from contributing clinical sites while keeping intact the relative timeline for the imaging examinations and clinical tests of each patient.

The MIDRC Grand Challenges Working Group (referred to as the “organizers”) recently conducted two grand challenges: the “COVIDx Challenge” followed by the “MIDRC mRALE Mastermind Challenge.” The COVIDx Challenge focused on the classification of portable chest radiographs (CXRs) as positive or negative for COVID-19, with COVID status for each case based on polymerase chain reaction (PCR) test results obtained from the patient no more than 2 days prior to acquisition of the CXR. This reference standard for the COVIDx Challenge relied on associated clinical data that accompanied the CXRs submitted to the MIDRC database; cases without PCR test results within 2 days prior to the corresponding portable CXR were not included. Subsequently, the MIDRC mRALE Mastermind Challenge fostered the development of artificial intelligence (AI) and machine learning (ML) techniques to address a more clinically relevant task: the assignment of mRALE (modified radiographic assessment of lung edema) scores^8^^–^^10^ (a score used to assess COVID severity level) to portable CXRs from patients known to have COVID-19 based on a recent positive PCR test. The purpose of this communication is to provide an overview of the MIDRC mRALE Mastermind Challenge and report the overall performance achieved by participants.

## 2 Materials and Methods

### 2.1 mRALE Score

**Fig. 1:**
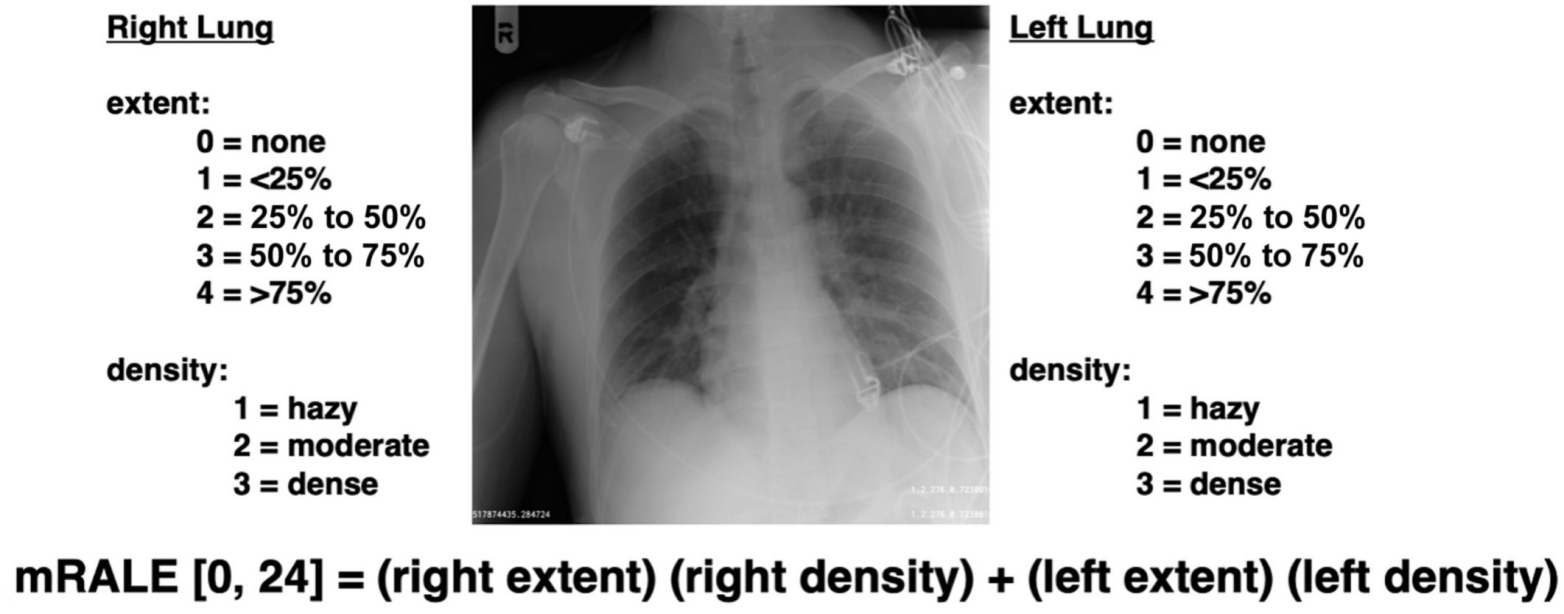
Portable CXR demonstrating mRALE score calculation.

The mRALE score is a variation of the RALE score, which was introduced to score chest radiographs of patients with acute respiratory distress syndrome (ARDS) as a “non-invasive measure that can be used to assess the severity of ARDS” and was found to be “independently associated with severity of ARDS as assessed by oxygenation and clinical outcomes including mortality.”^8^ Although the RALE score is computed across quadrants of a CXR, the mRALE score captures the same semi-quantitative information but across both lungs in the CXR rather than dividing the CXR into quadrants.^9^ In mRALE, an “extent of consolidation” score ranging from 0 to 4 (0, none; 1, <25%; 2, 25% to 50%; 3, 50% to 75%; 4, >75%) and a “density” score ranging from 1 to 3 (1, hazy; 2, moderate; 3, dense) are assigned by a radiologist to each lung; the products of the “extent of consolidation” and “density” scores for each of the two lungs are summed to obtain the CXR’s mRALE score (Fig. 1), which ranges from 0 to 24 (with higher scores reflecting greater severity of disease).

### 2.2 Cohorts

The training set for the MIDRC mRALE Mastermind Challenge was obtained from the public MIDRC data commons, and participants were allowed to use other data as well in the training of their algorithms. The training set included all imaging exams in the MIDRC public data commons available when this challenge was first organized that satisfied the inclusion criteria (adult patients with imaging performed no more than 1 day prior to and within 2 days after a positive COVID test). In this training set, multiple portable CXR exams per patient were allowed, and there were no specifications for patient demographics or disease severity distributions.

The validation and test sets were sampled from incoming data batches that had not yet been made publicly available. Inclusion criteria were patients age 18 years and older and imaging performed no more than 1 day prior to and within 2 days after of a positive COVID test. Cases in the validation and test sets comprised a single portable CXR per patient; if a patient had multiple portable CXRs, the CXR temporally closest to the PCR test was used. Moreover, to ensure that the validation and test cohorts used in the Challenge were representative of clinically relevant case distributions that would be encountered in the real world (and thus to minimize selection bias), cases were selected so that the distributions of patient age, sex at birth, and race/ethnicity in the validation and test sets approximated the distributions of these demographic factors from the United States Centers for Disease Control and Prevention (CDC) COVID-19 positive case distributions^11^; sampling of the available withheld cases (i.e., cases that had not yet been made publicly available) to achieve these approximate distributions was performed using a publicly available MIDRC-developed algorithm.^12^ There were no case-selection criteria based on medical center geographic location, patient socioeconomic status, or imaging equipment specifications, although some of these factors could be considered in future MIDRC challenges.

### 2.3 Reference Standard

**Fig. 2:**
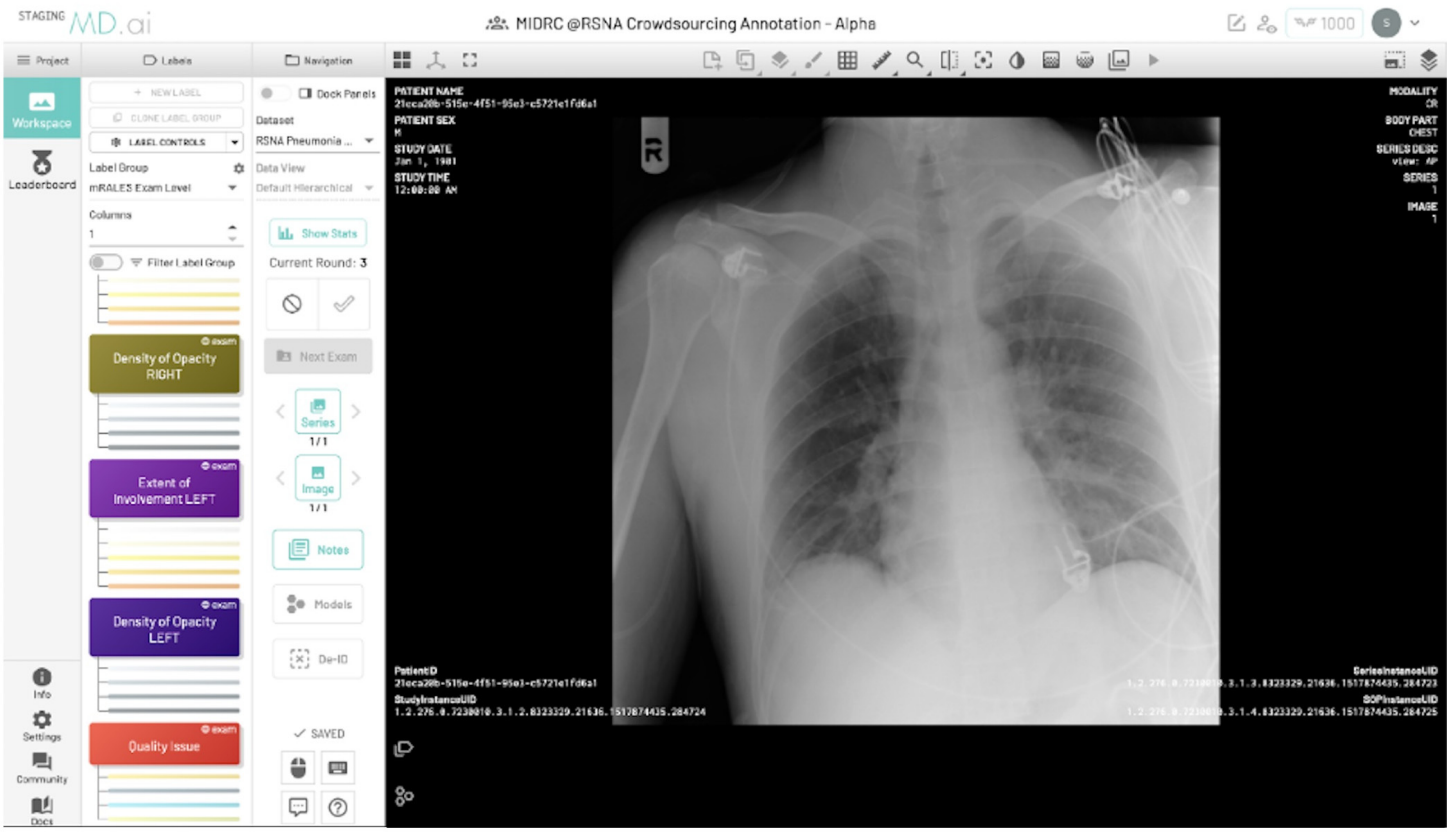
Interface designed to capture reference standard mRALE scores from the radiologist annotators.

Unlike the reference standard for the first MIDRC challenge (the COVIDx Challenge), which was based on clinical data that accompanied images in the MIDRC database, the mRALE scores that served as the reference standard for the MIDRC mRALE Mastermind Challenge did not already exist within MIDRC. The mRALE scores for the training, validation, and test sets were provided by 22 volunteer radiologist annotators who annotated each CXR with an appropriate mRALE score. Using software created by MD.ai,^13^ an interface (Fig. 2) was designed to efficiently capture mRALE scores from the annotators, who were radiologists solicited from among MIDRC collaborating sites and through professional societies.

Prospective annotators viewed a training video on the features of the MD.ai interface and the mRALE scoring process. Each prospective annotator then participated in a qualification task in which they were presented with 10 calibration cases through the MD.ai interface and asked to provide “extent of consolidation” and “density” scores for each lung, from which that annotator’s mRALE score was calculated for each case. The resulting mRALE scores of each annotator (along with the constituent “extent of consolidation” and “density” scores for each lung) were compared with the respective scores that had been established previously by a MIDRC researcher, a subspeciality chest radiologist with over 15 years of experience [CW] who had extensive knowledge of mRALE scoring. Only prospective annotators with fewer than 20% of their individual extent of consolidation or density scores differing by more than one from the respective established scores were able to serve as annotators for the Challenge.

The resulting pool of 22 radiologist annotators (half of whom were subspeciality chest radiologists) used the MD.ai annotation platform to asynchronously view the Challenge cases, verify image quality, and assign per-lung “extent of consolidation” and “density” scores, from which mRALE scores were automatically computed. Cases identified by an annotator as having poor image quality or poor patient positioning were removed from the Challenge datasets. Annotators logged in to the MD.ai system whenever they were available and annotated as many cases as they had time to complete over multiple sessions; the system randomly assigned cases to annotators until all cases had the requisite number of annotations. All cases in the training set received a single annotation, whereas all cases in the validation and test sets received annotations from three different annotators. The reference standard mRALE score assigned to each validation and test case was the mRALE score computed from the median “extent of consolidation” score and the median “density” score for each lung across the three annotators. This dual approach to the reference standard (singly annotated training cases and triply annotated validation and test cases) was meant to balance the efficiency of annotator time with the desire to capture annotator variability for the cases against which participants’ algorithms would be evaluated.

After annotations were obtained for all cases, the distributions of reference mRALE scores and patient age, sex, race, and ethnicity were matched across the validation and test sets using a publicly available stratified sampling algorithm customized for this purpose.^14^^,^^15^ This procedure ensured that performance on the validation set was a fair indication of the expected performance on the test set and that patient demographics matched those of the CDC case distributions to within 5%. This approach, however, meant that not all cases intended for the validation and test sets were eventually used in the Challenge because of deviations from the desired distributions in mRALE score (i.e., COVID severity) and in patient demographics.

### 2.4 Performance Assessment

The main performance metric used to rank the AI/ML algorithms of Challenge participants was the quadratic-weighted kappa statistic^16^ between the algorithm-estimated mRALE scores and the reference mRALE scores. The secondary performance metric was the prediction probability concordance,^17^ which was used to break any ties. Although a statistically significant difference in performance among algorithms was not required to win the Challenge, *a posteriori* bootstrapping (random sampling with replacement for 1000 iterations) was performed to assess whether differences in performance between the winning algorithm and the other algorithms were statistically significant. A correction for performing multiple comparisons was performed using Holm-Bonferroni.

Agreement between the reference standard mRALE scores and the mRALE scores output by the participants’ algorithms and agreement among the outputs of the different algorithms were analyzed; 95% confidence intervals for the reported quadratic-weighted kappa statistic values were computed through *a posteriori* bootstrapping. In instances where superiority could not be established, equivalence testing was conducted with an equivalence margin set at 0.01 for the quadratic-weighted kappa statistic. In addition, to explore whether combining the output of different algorithms could improve performance beyond that of the winning algorithm, the outputs of the top-ranked n algorithms (where the value of n was allowed to range from two to the total number of submitted algorithms) were combined by obtaining the (1) median, (2) minimum, and (3) maximum values across the n outputs. Furthermore, a secondary performance assessment was conducted by excluding those cases with a reference mRALE score of zero to determine whether the estimated performance levels were affected by the presence of CXRs that demonstrated no apparent lung involvement.

To relate the performance of participants’ algorithms to the real-world complexity of the task of assigning mRALE scores to portable CXRs, the quadratic-weighted kappa statistic was computed among the three sets of annotations assigned to the test set cases. Although the three annotations per case were not provided by the same three annotators across all test set cases, the arbitrarily assigned “Annotation 1,” “Annotation 2,” and “Annotation 3” for all test set cases were compared in a pair-wise manner using the quadratic-weighted kappa statistic. This assessment of agreement among annotation sets offered insight into the variability among radiologists in the task of assigning mRALE scores.

### 2.5 Challenge Logistics

The Challenge was hosted on the MedICI challenge platform,^18^^,^^19^ which was configured to accept Docker container submissions during the validation and test phases. Accordingly, participants did not have access to the cases in the validation and test sets; instead, their algorithms were run on the challenge platform by the organizers against the validation and test sets in the validation and test phases, respectively.

The training phase of the Challenge began on April 26, 2023. The training set of cases that had been identified in the public MIDRC data commons and annotated with mRALE scores was made known to Challenge participants, and the associated annotator-defined reference mRALE scores for these cases were released. Participants were free to use any other cases to which they had access for training, but reference mRALE scores were only provided for the cases in the organizer-provided training set. Participants had an opportunity to practice Docker submissions through the MedICI platform during this phase, and any such submissions were run against a small set of 12 “practice” cases on the platform.

The validation phase of the Challenge began on June 10, 2023. During this phase, participants submitted Docker containers of their trained algorithms and received feedback on the performance of their algorithms when applied to the sequestered cases in the validation set. The performance of participants’ models (for both performance assessment metrics) was posted to a leaderboard.

The test phase of the Challenge was open from July 1 to 10, 2023. During this phase, participants (individuals or teams) submitted Docker versions of their final trained and validated algorithms, which were run on the test set. Each participant was allowed to submit up to three different trained algorithms, with only the best-performing algorithm used for final ranking and analysis. Participants were not informed of the performance of any of their methods during the test phase until the conclusion of the Challenge, at which time results were released on the leaderboard with the final rankings of all algorithms.

As an incentive to increase interest in the Challenge, the NIBIB provided cash awards for the seven highest-ranked participants (first place: $15,000; second place: $8000; third place: $7000; fourth through seventh places: $5000 each). In addition, the two highest-ranked participants were offered assistance from MIDRC to take their algorithms through the FDA regulatory process. Monetary prizes were contingent on the AI/ML algorithm outperforming random guessing and on participants depositing their algorithms on the MIDRC public GitHub repository for the benefit of the medical imaging research community. MIDRC has established an explicit conflict of interest policy, which states that an individual (or anyone who directly reports to that individual) may not competitively participate in a MIDRC challenge if the individual (1) was involved with any aspects of the organization of the challenge; (2) had detailed knowledge of, or access to, any test data used in the challenge prior to all challenge participants; (3) had any other advance knowledge pertaining to the challenge before it was available to all challenge participants; or (4) participated in the annotation of test set cases.

## 3 Results

### 3.1 Cohorts

**Fig. 3:**
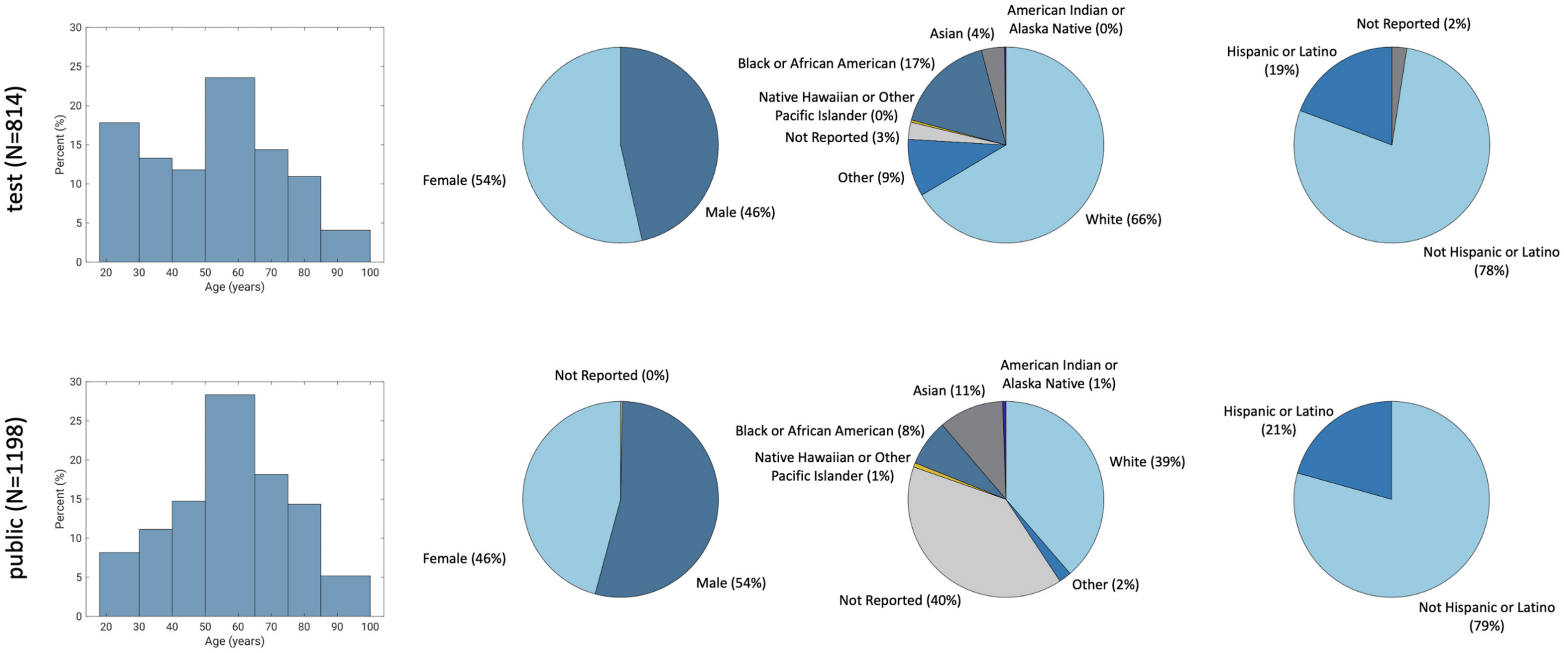
Demographic distributions of the test set cases (n=814) in terms of age groups, sex, race, and ethnicity.

In the public MIDRC data commons available at the time, 2115 imaging studies from 1234 patients satisfied the inclusion criteria; 36 of these cases were disqualified due to image quality issues identified during the annotation process, resulting in a training set that consisted of 2079 singly annotated portable CXRs. From among cases in the incoming data batches reserved for challenge validation and test sets, 1278 cases satisfied the inclusion criteria; 11 of these cases were disqualified due to image quality issues identified during the annotation process, resulting in 1267 cases being triply annotated for use in either the validation or test sets. Stratified sampling for distribution matching^14^ eliminated 256 of these cases so that the final validation and test sets consisted of 197 and 814 portable CXRs, respectively. Figure 3 captures the demographic distributions of the Challenge training and test cases.

### 3.2 Reference Standard

**Fig. 4:**
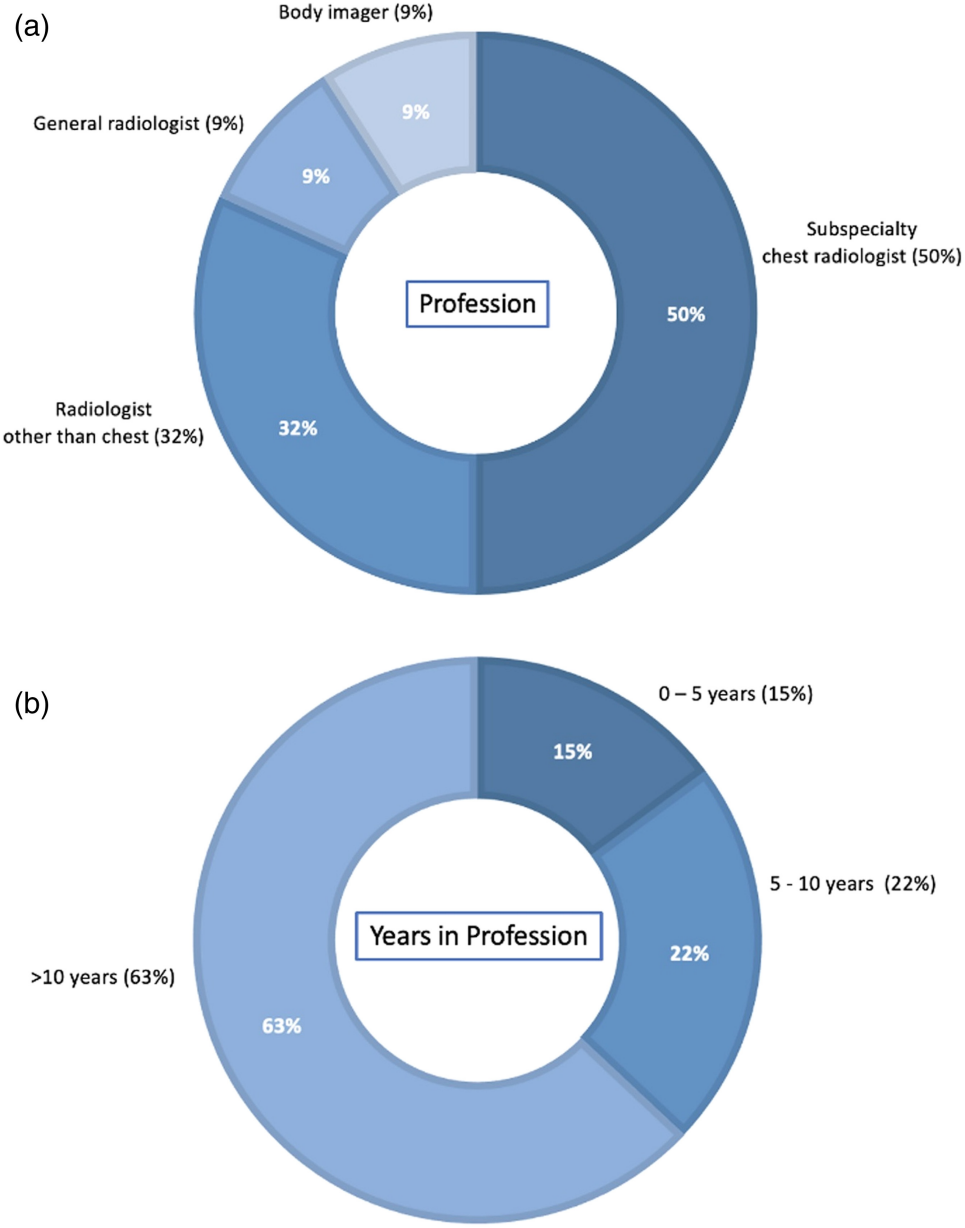
Distribution of experience of the 22 annotators who provided the mRALE score reference standard in terms of (a) specialty and (b) years in the profession.

**Fig. 5:**
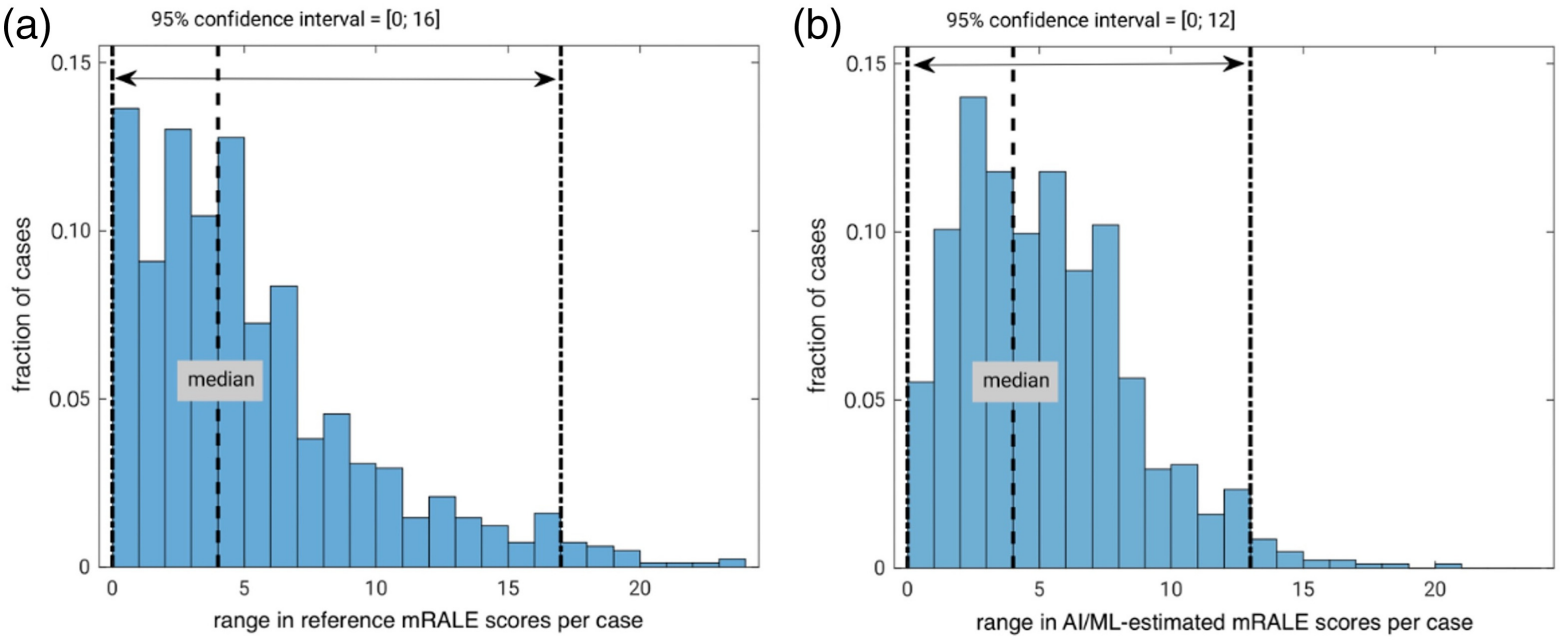
Histograms capturing the range in mRALE scores for cases in the test set (n=814) obtained from (a) the reference standard (three annotations per case) and (b) the output provided across all nine participants’ algorithms.

**Fig. 6:**
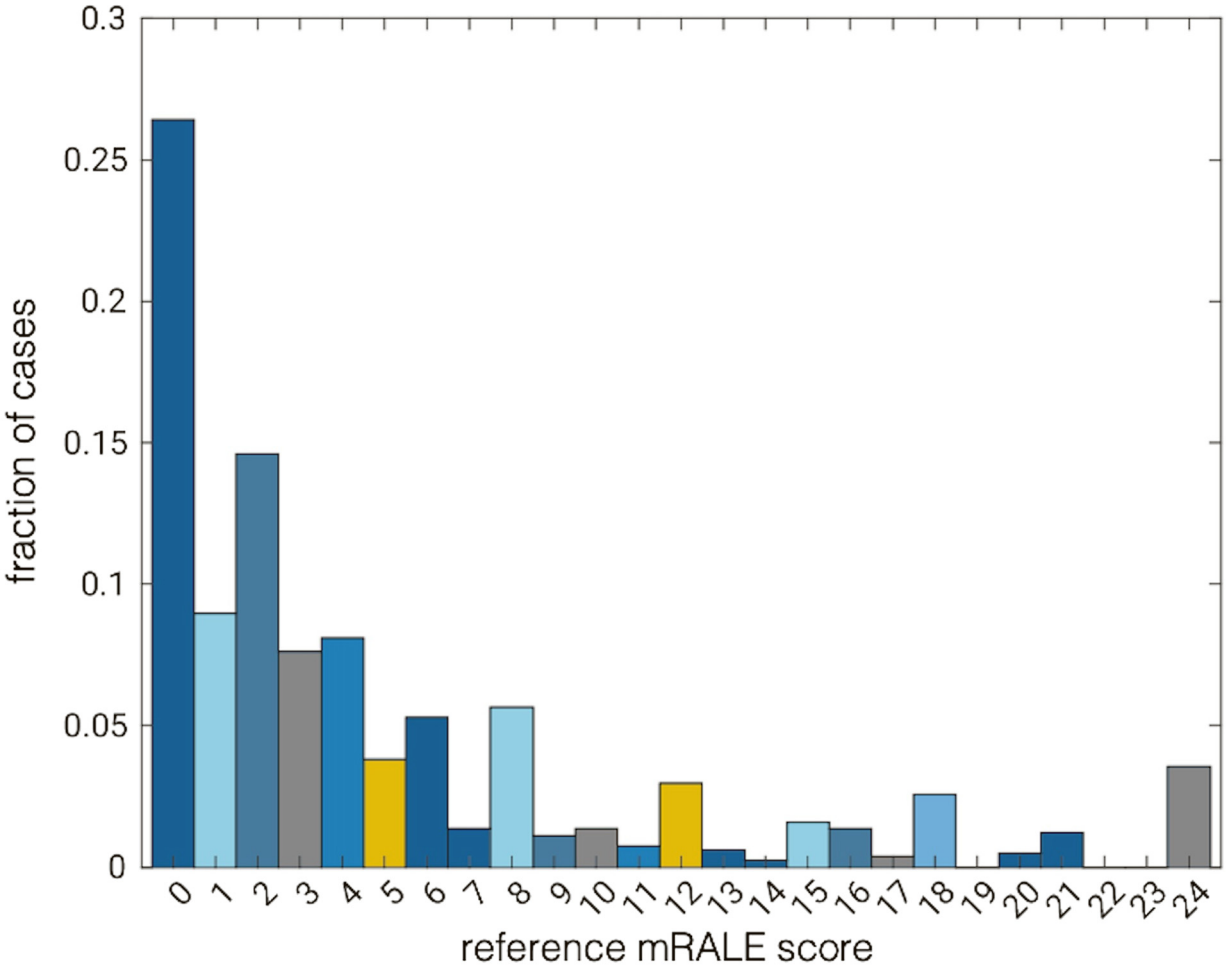
Distribution of reference mRALE scores for cases in the test set (n=814). Note that by definition mRALE scores of 19, 22, and 23 are not possible. Colors are intended as a visual guide.

The experiences of the 22 radiologist annotators who created the Challenge reference standard are presented in Fig. 4. The number of training, validation, and test cases annotated by any one annotator ranged from 2 to 1163 (median: 170 cases per annotator). There was considerable variability in annotator-assigned mRALE scores for the test set cases (which were triply annotated) [Fig. 5(a)]: the quadratic-weighted kappa statistic across the three annotation sets ranged from 0.68 to 0.74. The reference mRALE was zero for 26% of the test cases, indicating no lung involvement despite all cases having had a positive COVID-19 test within 2 days of the CXR exam (Fig. 6).

### 3.3 Submitted AI/ML Algorithms

Submitted algorithms had been trained using various sources of data, including the publicly available and annotated training set from MIDRC (described previously), the RSNA Pneumonia Detection Challenge dataset,^20^ and other datasets such as CheXpert^21^ and TorchXRayVision.^22^ Some submissions incorporated manually curated private datasets. Some participants applied additional elements of quality assurance to the MIDRC training set, including the selection of specific images from each case, removal of images considered by the participant to be of poor quality, and manual annotations to improve data utility.

In terms of general methodology, several algorithms employed sequential pretraining and fine-tuning on external datasets to leverage existing knowledge. The majority of submitted algorithms involved the use of convolutional neural networks (CNNs) for feature extraction and prediction. Data preprocessing, augmentation, and post-processing techniques were employed by all algorithms to enhance model performance and generalization. Training strategies varied in terms of model architectures, loss functions, optimization algorithms, and data augmentation techniques. Although some algorithms employed ensemble methods to combine predictions from multiple models, other algorithms focused on training individual models with specific objectives. Algorithms also differed in terms of cross-validation strategies, with some utilizing k-fold cross-validation and others employing holdout validation.

Overall, the algorithms submitted to the test phase of the Challenge demonstrated a diversity of approaches to address the complexities of AI prediction of COVID-19 severity on portable CXRs using mRALE scores. Descriptions of the methods can be found in the MIDRC GitHub repository.^23^

### 3.4 AI/ML Performance

The first-place participant was a team led by Ian Pan, M.D. from Brigham and Women’s Hospital, the second-place participant was a team led by Ran Zhang, Ph.D. from the University of Wisconsin-Madison, and the third-place participant was a team led by Finn Behrendt from the Hamburg University of Technology.

**Fig. 7:**
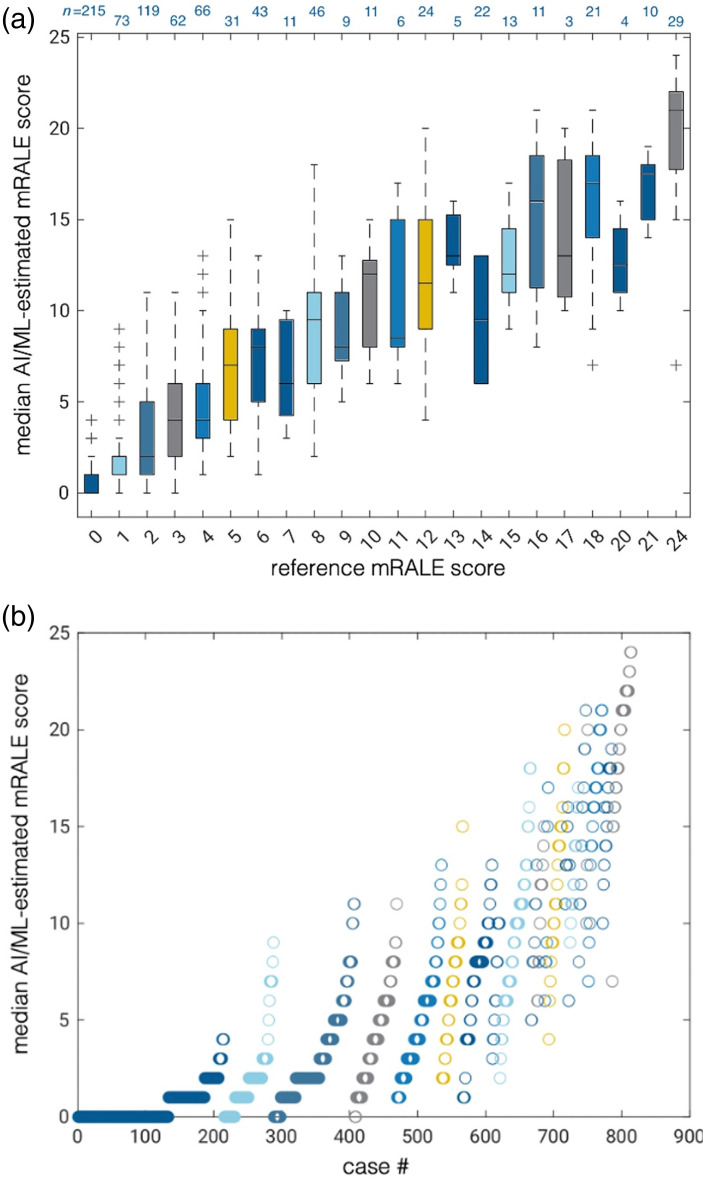
(a) Box-and-whiskers plot of the per-case median mRALE scores across the nine submitted algorithms for each reference mRALE score for cases in the test set (n=814) and (b) scatter plot of the per-case median mRALE scores across the nine submitted algorithms for all cases in the test set, with cases ordered from low to high reference mRALE score. Note that by definition mRALE scores of 19, 22, and 23 are not possible. Colors are intended as a visual guide.

**Fig. 8:**
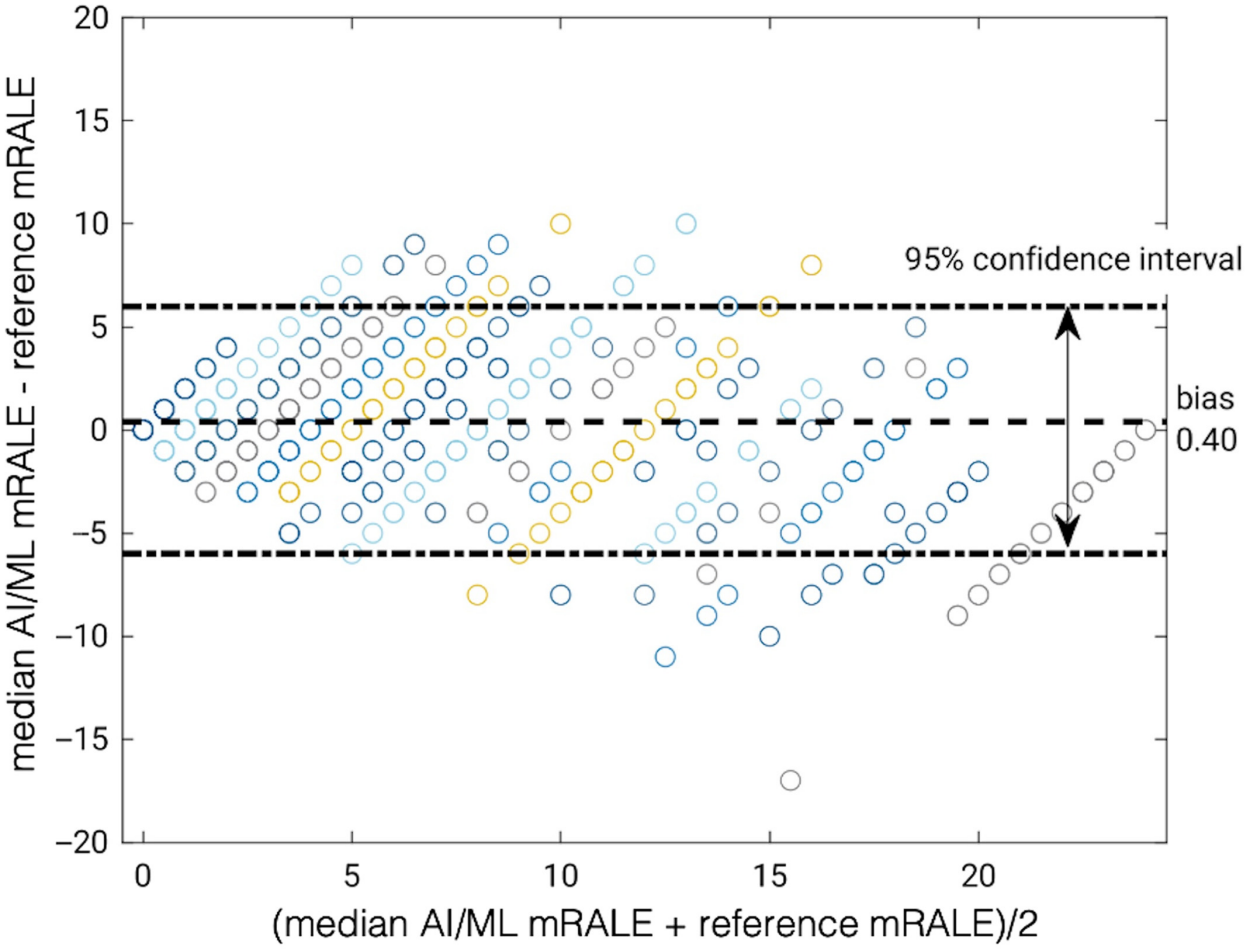
Bland-Altman plot of the reference standard mRALE scores and the median of the mRALE scores across the nine participants’ algorithms for the test set (n=814). Cases are color-coded in the same way as in Fig. 6 as a visual guide.

There was considerable variability in mRALE scores estimated by the nine participants’ algorithms for each case in the test set [Fig. 5(b)]. The median of the mRALE scores across the nine algorithms for each case correlated with the reference standard mRALE score, although a wide range in differences with the reference standard was observed (Fig. 7). A Bland-Altman plot (Fig. 8) illustrates that there was a minimal bias of 0.40 between the median algorithm-estimated mRALE scores and the reference mRALE scores with limits of agreement of ±6; however, the AI/ML algorithms tended to underestimate COVID-19 severity for cases with higher reference mRALE scores. A comparison of the mRALE scores for each of the nine submitted AI/ML algorithms to the reference standard across the test set cases yielded similar results (Figs. S1–S9 in the Supplementary Material).

**Fig. 9:**
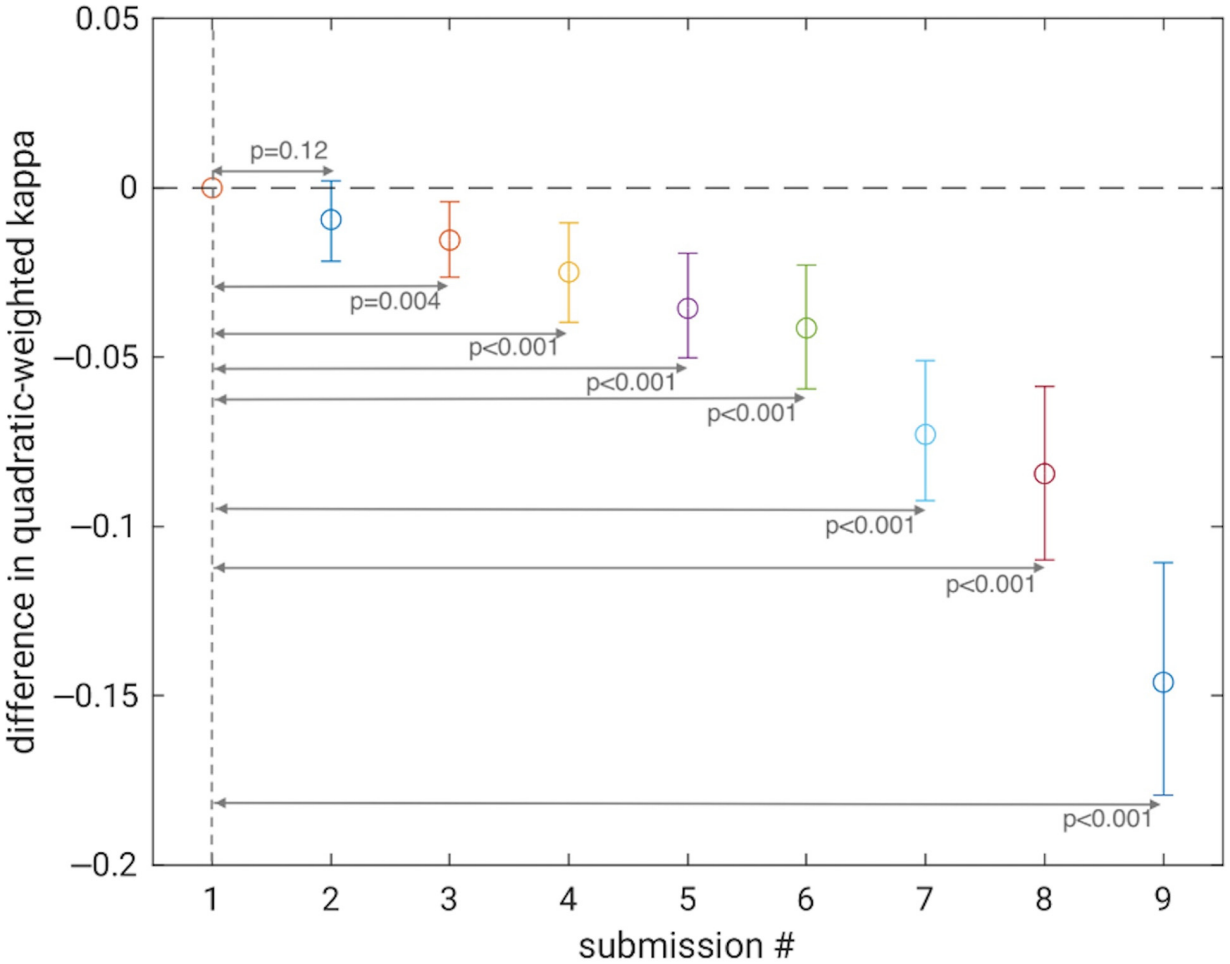
Difference in quadratic-weighted kappa values between the winning algorithm (submission #1, quadratic-weighted kappa 0.885 [0.862 to 0.904]) and the other algorithms (appearing in the same order as in Table 1), with error bars indicating the 95% confidence intervals.

The quadratic-weighted kappa of the agreement between the reference mRALE scores and the mRALE scores estimated by the nine participants’ algorithms ranged from 0.739 (95% confidence interval [0.693, 0.777]) to 0.885 [0.862; 0.904] (Table 1). The ordering of the submitted algorithms by prediction probability concordance (the secondary performance assessment metric) was almost identical to the ordering of the algorithms by quadratic-weighted kappa (the primary performance metric). The difference in quadratic-weighted kappa between the winning submission and the second-place submission failed to reach statistical significance (p=0.12), but differences between all other submissions and the winning submission were statistically significant, with p-values <0.004 remaining statistically significant after correcting for multiple comparisons (Fig. 9). The performance of the second-place algorithm was not equivalent to that of the winner within the equivalence margin of 0.01 (Fig. 9).

**Table 1:** Quadratic-weighted kappa statistic and prediction probability concordance (with 95% confidence intervals (CIs)) for the nine participants’ algorithms assessing agreement between the algorithm-estimated mRALE scores and the reference standard mRALE scores for the test set (n=814). Participants are numbered in order of decreasing kappa value, which represents the final rank order in the Challenge.

Participant	Quadratic-weighted kappa (95% CI)	Prediction probability concordance (95% CI)
1	0.885 [0.862, 0.904]	0.875 [0.863, 0.887]
2	0.875 [0.854, 0.895]	0.876 [0.865, 0.888]
3	0.869 [0.846, 0.889]	0.875 [0.862, 0.888]
4	0.859 [0.835, 0.881]	0.861 [0.848, 0.874]
5	0.849 [0.821, 0.872]	0.858 [0.845, 0.871]
6	0.843 [0.813, 0.868]	0.853 [0.839, 0.866]
7	0.812 [0.780, 0.839]	0.842 [0.829, 0.856]
8	0.799 [0.767, 0.830]	0.820 [0.806, 0.835]
9	0.739 [0.693, 0.777]	0.811 [0.794, 0.828]

Notably, the quadratic-weighted kappa statistic values among the nine submitted algorithms on the test set ranged from 0.751 to 0.971, indicating apparently higher agreement among the AI/ML-estimated mRALE scores than among the three sets of annotations provided by the 22 radiologist annotators. Combining the output from the AI/ML algorithms by taking the median mRALE score across all nine submitted algorithms for each case resulted in a quadratic-weighted kappa of 0.879 [0.856, 0.900] when compared with the reference mRALE scores. None of the combinations of algorithms (top 2 algorithms, top 3 algorithms, etc.) outperformed the winning submission; algorithm combinations, however, were equivalent in performance (within an equivalence margin of 0.01 for the quadratic-weighted kappa statistic) when up to the top 5 algorithms were combined, beyond which equivalence was no longer demonstrated. Similar results were obtained when the output of the algorithms was combined using the per-case minimum or maximum of the algorithms’ mRALE scores; with the minimum-score approach, equivalence could only be established when combining the winning and second-place algorithms, whereas no equivalence was established for the maximum-score approach.

In the secondary performance analysis that excluded cases with a reference mRALE score of zero, similar results were obtained but with small, statistically significant decreases in performance. In this analysis, the winning algorithm achieved a quadratic-weighted kappa statistic of 0.851 [0.820, 0.877] (p<0.001 compared with its performance of 0.885 with all test set cases). The ranking of algorithms remained consistent, with the decrease in performance slightly increasing for lower-ranked algorithms. The largest drop in quadratic-weighted kappa statistic value was −0.058 [−0.0745, −0.044].

## 4 Discussion

The MIDRC mRALE Mastermind Challenge introduced a framework for evaluating AI systems in the assessment of COVID-19 severity from portable CXRs. To ensure the integrity and reproducibility of the findings, challenge organizers required participants to submit containerized models for centralized evaluation on sequestered validation and test cases.^24^ Although imposing a computational burden on the organizers, this approach ultimately enhanced the quality and reliability of the outcomes; by preventing potential manipulation of results and preserving the integrity of the validation and test sets, participants were unable to “train to the test,” thus bolstering the translational potential of the findings.^2^

The large proportion of cases (26%) without lung involvement (mRALE = 0), despite being COVID-positive, highlights the challenge of diagnosing COVID-19 based solely on CXRs; although the objective of this study was to assess COVID severity rather than diagnose COVID positivity, the prevalence of cases without lung involvement certainly influenced the reported performances, resulting in a statistically significant increase in performance compared with performance when cases without lung involvement were excluded. Consideration of additional performance assessment metrics may result in a more comprehensive understanding of the potential for AI/ML algorithms in this important clinical task.

Although statistical significance in performance differences between submitted algorithms was not a consideration for algorithm ranking, it was an important part of the subsequent evaluation. Although some algorithms exhibited higher agreement with the radiologist annotators of the test set in this challenge, variations in performance across diverse datasets and clinical settings remain to be explored. Furthermore, the question of whether the observed performance differences that reached statistical significance hold clinical significance is beyond the scope of this manuscript.

The primary limitation of this study was that the reference standard mRALE scores for COVID-19 severity relied on human expert opinion, which was highly variable among radiologist annotators; the use of the median mRALE score across the three annotations per case served to decrease the impact of this variability on the overall results. Another limitation was that the distributions of patient demographics, including age group, sex, race, and ethnicity, were matched to CDC distributions for COVID-positive cases; however, using distributions for patients hospitalized for COVID-19 may have provided a more representative sample of the population undergoing portable chest radiography for COVID-19 assessment. Thus, despite the measures taken to preserve the integrity of the validation and test sets, biases cannot be completely eliminated, which may yield disparities in model performance in future settings.

The Challenge results offer insights into the utility and limitations of medical image analysis with AI algorithms in a research setting, which could help inform potential clinical translation and regulatory approval. The findings highlight the ongoing challenge of achieving accurate and reliable predictions of COVID-19 severity from CXRs, both for human experts and AI/ML methods. Future research efforts should prioritize addressing biases and improving model generalization. The lessons learned from the MIDRC mRALE Mastermind Challenge are guiding the design and execution of other MIDRC challenges, including the MIDRC XAI Challenge, which was recently completed and focused on explainable AI to assess the extent of lung involvement in the CXRs of COVID-positive patients.

## 5 Conclusion

The MIDRC mRALE Mastermind Challenge introduced a framework for evaluating AI systems in the assessment of COVID-19 severity from portable CXRs and, through the efforts of the groups that participated, revealed the potential of AI in this task as evidenced by promising performance against the reference standard. The observed variability in the reference mRALE scores highlights the challenges in standardizing severity assessment. These findings contribute to ongoing efforts to develop AI technologies for potential use in clinical practice and offer insights for the enhancement of COVID-19 severity assessment.

## Supplementary Material

10.1117/1.JMI.12.2.024505.s01

## Data Availability

The images used in the MIDRC mRALE Mastermind Grand Challenge are publicly available in the MIDRC Data Commons at data.midrc.org

## References

[1] MIDRC, “Medical imaging and data resource center,” https://www.midrc.org/ (accessed 1 March 2024).

[2] Armato S. G. III Drukker K. Hadjiiski L., “AI in medical imaging grand challenges: Translation from competition to research benefit and patient care,” Br. J. Radiol. 96, 20221152 (2023). BJRAAP 0007-1285 10.1259/bjr.20221152 37698542 PMC10546459

[3] El Naqa I. et al., “Lessons learned in transitioning to AI in the medical imaging of COVID-19,” J. Med. Imaging 8(suppl. 1), 010902 (2021). JMEIET 0920-5497 10.1117/1.JMI.8.S1.010902 PMC8488974 34646912

[r4] Rouzrokh P. et al., “Mitigating bias in radiology machine learning: 1. Data handling,” Radiol. Artif. Intell. 4, e210290 (2022). 10.1148/ryai.210290 36204544 PMC9533091

[r5] Zhang K. et al., “Mitigating bias in radiology machine learning: 2. Model development,” Radiol. Artif. Intell. 4, e220010 (2022). 10.1148/ryai.220010 36204532 PMC9530765

[6] Faghani S. et al., “Mitigating bias in radiology machine learning: 3. Performance metrics,” Radiol. Artif. Intell. 4, e220061 (2022). 10.1148/ryai.220061 36204539 PMC9530766

[7] U.S. Department of Health and Human Services, (§164.514(b)(2)), https://www.hhs.gov/hipaa/for-professionals/privacy/special-topics/de-identification/index.html (accessed 4 May 2024).

[8] Warren M. A. Zhao Z. Koyama T., “Severity scoring of lung oedema on the chest radiograph is associated with clinical outcomes in ARDS,” Thorax 73, 840–846 (2018). THORA7 0040-6376 10.1136/thoraxjnl-2017-211280 29903755 PMC6410734

[r9] Li M. D. et al., “Automated assessment and tracking of COVID-19 pulmonary disease severity on chest radiographs using convolutional Siamese neural networks,” Radiol. Artif. Intell. 2(4), e200079 (2020). 10.1148/ryai.2020200079 33928256 PMC7392327

[10] Li M. D. et al., “Multi-radiologist user study for artificial intelligence-guided grading of COVID-19 lung disease severity on chest radiographs,” Acad. Radiol. 28(4), 572–576 (2021). 10.1016/j.acra.2021.01.016 33485773 PMC7813473

[11] Centers for Disease Control and Prevention, “COVID data tracker,” https://covid.cdc.gov/covid-data-tracker/#datatracker-home (accessed 21 March 2022).

[12] MIDRC, “Task-based-sampling,” https://github.com/MIDRC/task-based-sampling (accessed 1 March 2024).

[13] Tsai E. B. et al., “The RSNA International COVID-19 Open Radiology Database (RICORD),” Radiology 299(1), E204–E213 (2021). RADLAX 0033-8419 10.1148/radiol.2021203957 33399506 PMC7993245

[14] Baughan N. et al., “Sequestration of imaging studies in MIDRC: stratified sampling to balance demographic characteristics of patients in a multi-institutional data commons,” J. Med. Imaging 10(6), 064501 (2023). JMEIET 0920-5497 10.1117/1.JMI.10.6.064501 PMC10704184 38074627

[15] MIDRC, “Stratified_sampling,” https://github.com/MIDRC/Stratified_Sampling (accessed 1 March 2024).

[16] McHugh M. L., “Interrater reliability: the kappa statistic,” Biochem. Med. 22(3), 276–282 (2012). 10.11613/BM.2012.031 PMC3900052 23092060

[17] Smith W. D. Dutton R. C. Smith N. T., “A measure of association for assessing prediction accuracy that is a generalization of non-parametric ROC area,” Stat. Med. 15(11), 1199–1215 (1996). SMEDDA 1097-0258 10.1002/(SICI)1097-0258(19960615)15:11<1199::AID-SIM218>3.0.CO;2-Y 8804148

[18] MedICI-NCI, “MedICI,” https://github.com/MedICI-NCI/MedICI (accessed 7 May 2024).

[19] Pavao A. et al., “CodaLab competitions: an open source platform to organize scientific challenges,” Ph.D. thesis, Université Paris-Saclay (2022).

[20] Prevedello L. M. et al., “Challenges related to artificial intelligence research in medical imaging and the importance of image analysis competitions,” Radiol. Artif. Intell. 1(1), e180031 (2019). 10.1148/ryai.2019180031 33937783 PMC8017381

[21] Irvin J. et al., “CheXpert: a large chest radiograph dataset with uncertainty labels and expert comparison,” Proc. AAAI Conf. Artif. Intell. 33(1), 590–597 (2019). 10.1609/aaai.v33i01.3301590

[22] Cohen J. P. et al., “TorchXRayVision: a library of chest X-ray datasets and models,” arXiv:2111.00595 (2021).

[23] MIDRC, “Medical imaging and data resource center,” https://github.com/midrc (accessed 3 May 2024).

[24] Guinney J. Saez-Rodriguez J., “Alternative models for sharing confidential biomedical data,” Nat. Biotechnol. 36, 391–392 (2018). NABIF9 1087-0156 10.1038/nbt.4128 29734317

