# Supplementary material for: MIDRC mRALE Mastermind Grand Challenge: AI to predict COVID severity on chest radiographs
Source: J Med Imaging (Bellingham). 2025 Apr 18;12(2):024505. doi: 10.1117/1.JMI.12.2.024505 (PMC12014941; doi:10.1117/1.JMI.12.2.024505)
Supplement: Supplementary file 1 [file JMI_012_024505_SD001.pdf]

Supplemental material

A. Challenge participants

**Team / Team Members**	**Affiliation**
1. Ian Pan, MD	Brigham and Women's
2. Ran Zhang, PhD	University of Wisconsin-Madison
3. Finn Behrendt	University of Technology Hamburg
4. Team MALTA:<br>Christian Mattjie<br>Luis Vinicius de Moura<br>Rafaela Cappelari Ravazio<br>Otavio Parraga<br>Lucas Silveira Kupssinskü, PhD<br>Rodrigo Coelho Barros, PhD	Pontificia Universidade Católica do Rio Grande do Sul
5. Yijie Yuan	Johns Hopkins Medicine
6. Team UKen:<br>Cohen Archbold<br>Imran Abdullah-Al-Zubaer, PhD<br>Atik Ahamed	University of Kentucky
7. Mathieu Goulet, PhD	Centre Régional Intégré de Cancérologie (CRIC)
8. Team Waterloo:<br>Yifan Wu[1]<br>Hayden Gunraj[1]<br>Chengzong Zhao[1]<br>Yuhao Chen, PhD[1]<br>Alexander Wong, PhD[1,2]<br>Pengcheng Xi, PhD[1,3]	[1] University of Waterloo<br>[2] Waterloo AI Institute<br>[3] National Research Council of Canada
9. Team Computational Health:<br>Stanley Liang, PhD<br>Sameer Antani, PhD<br>Zhiyun Xue, PhD<br>Sivaramakrishnan Rajaraman, PhD<br>Feng Yang, PhD	Research Branch (CHRB), National Library of Medicine (NLM) / NIH

Table S1. Participants in the MIDRC mRALE Mastermind Challenge.

B. Additional figures

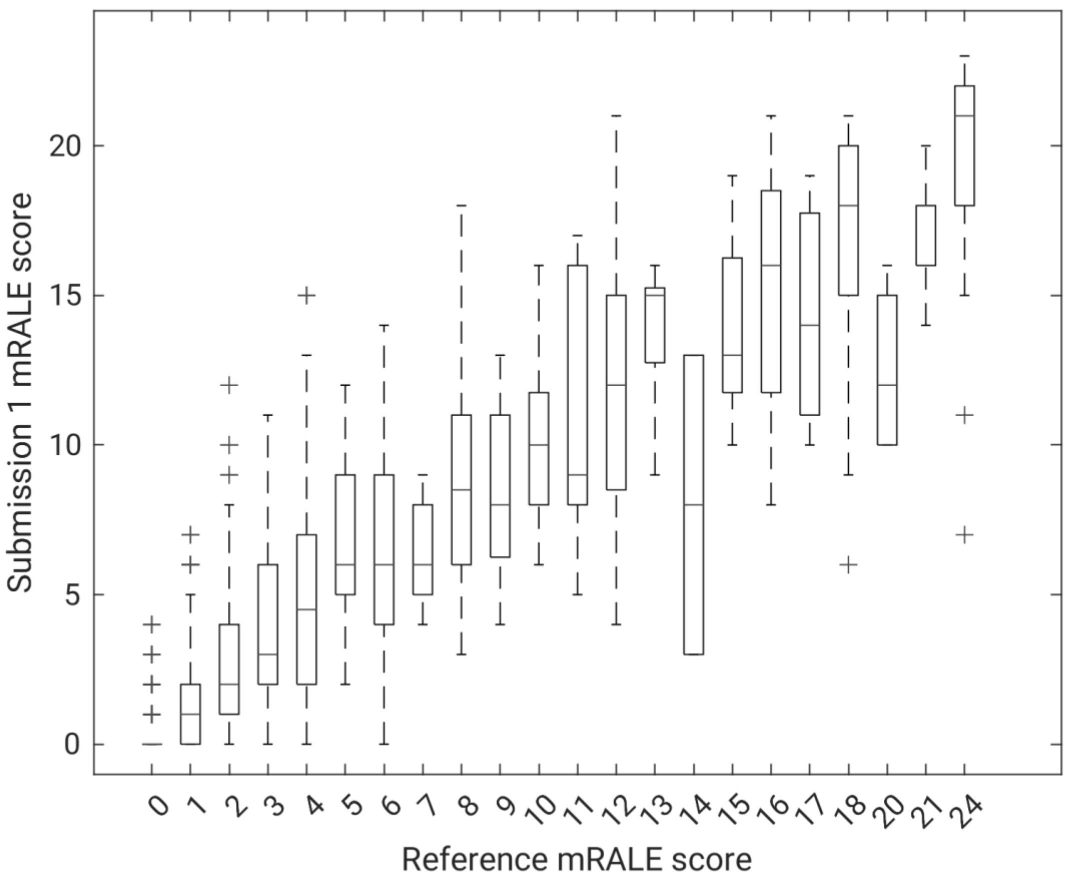

Figure S1. Box-and-whiskers plot for Submission 1 capturing the range of mRALE scores output by the algorithm for the test set cases across the range of test set reference mRALE score.

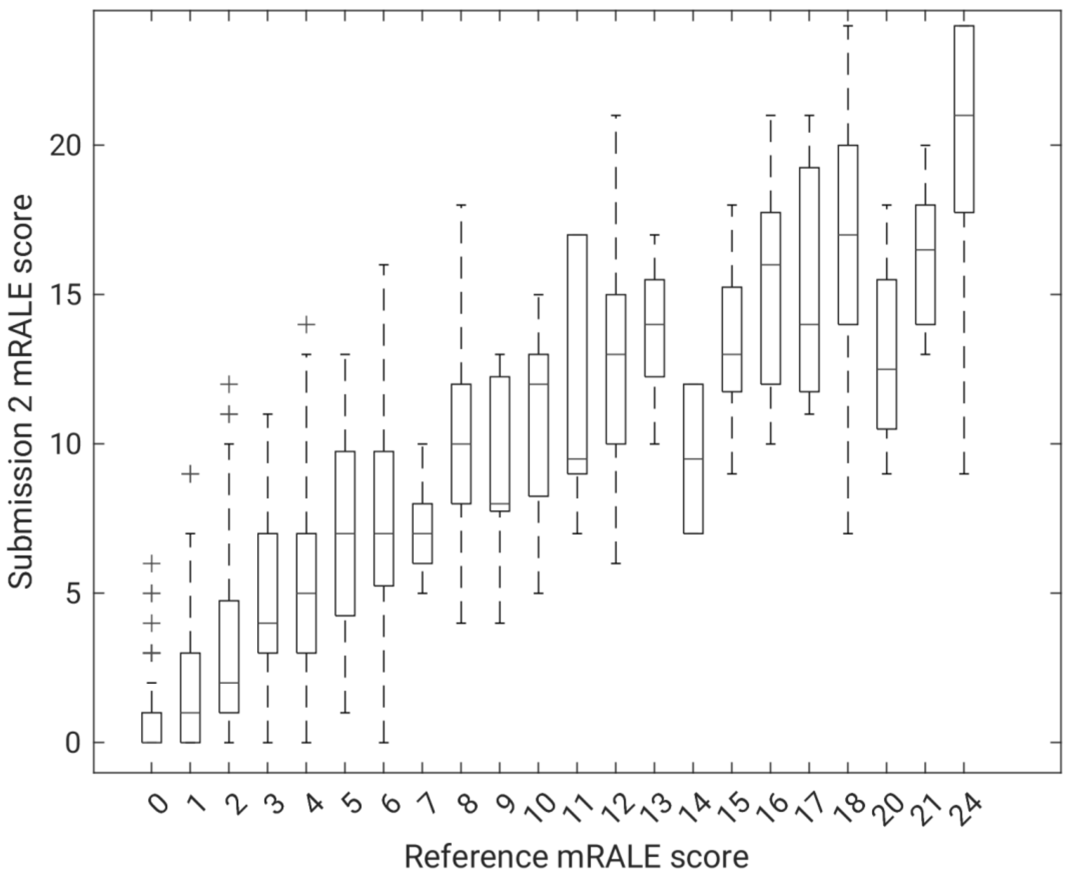

Figure S2. Box-and-whiskers plot for Submission 2 capturing the range of mRALE scores output by the algorithm for the test set cases across the range of test set reference mRALE score.

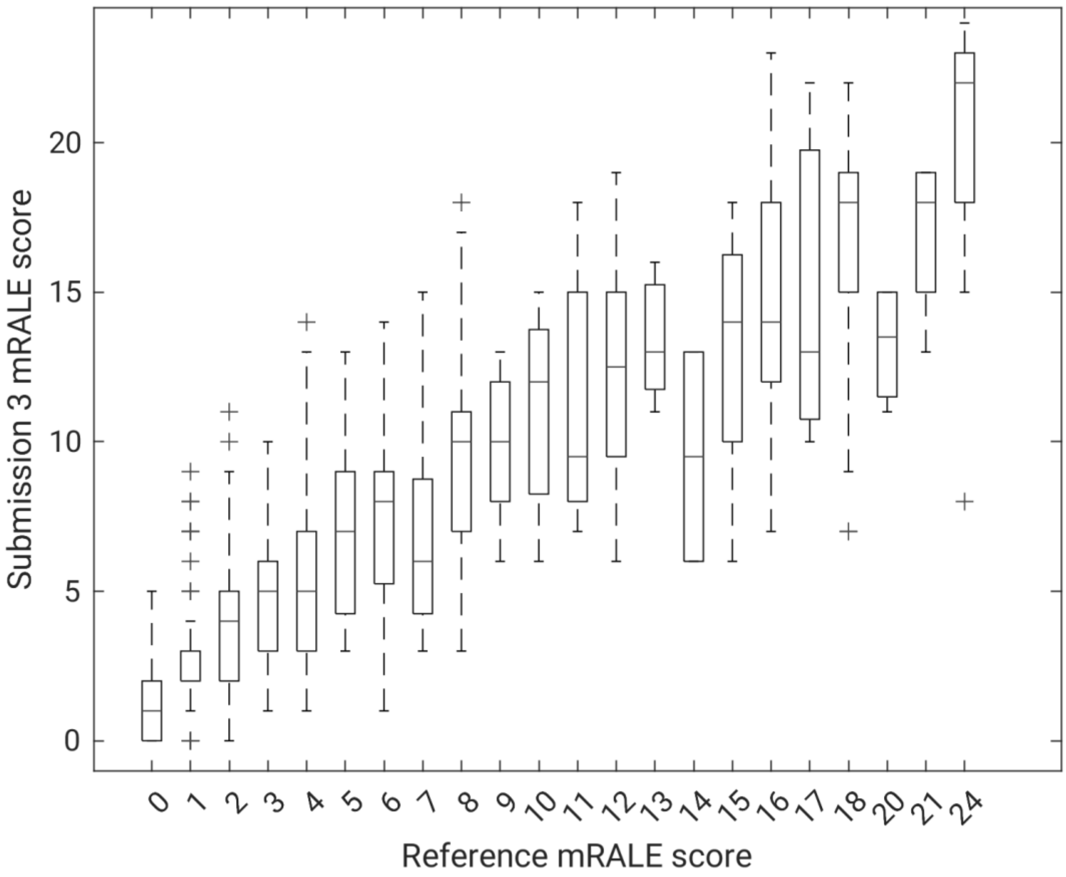

Figure S3. Box-and-whiskers plot for Submission 3 capturing the range of mRALE scores output by the algorithm for the test set cases across the range of test set reference mRALE score.

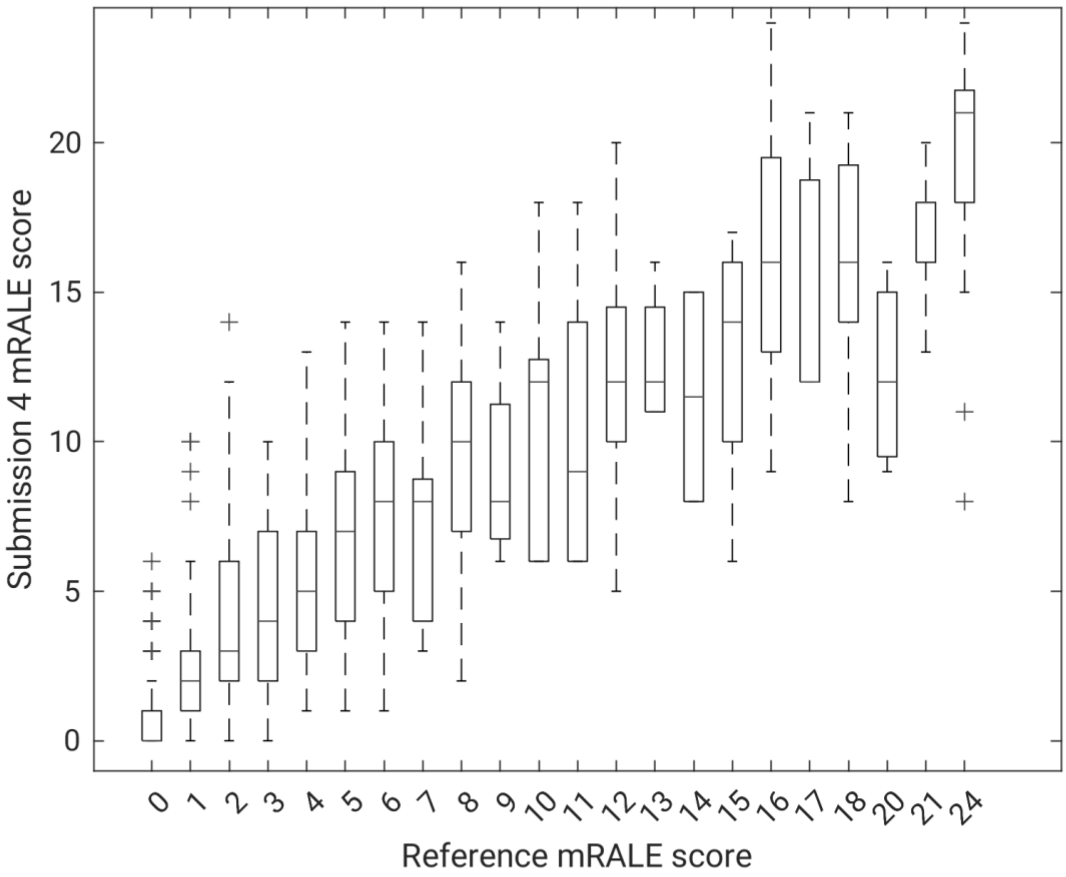

Figure S4. Box-and-whiskers plot for Submission 4 capturing the range of mRALE scores output by the algorithm for the test set cases across the range of test set reference mRALE score.

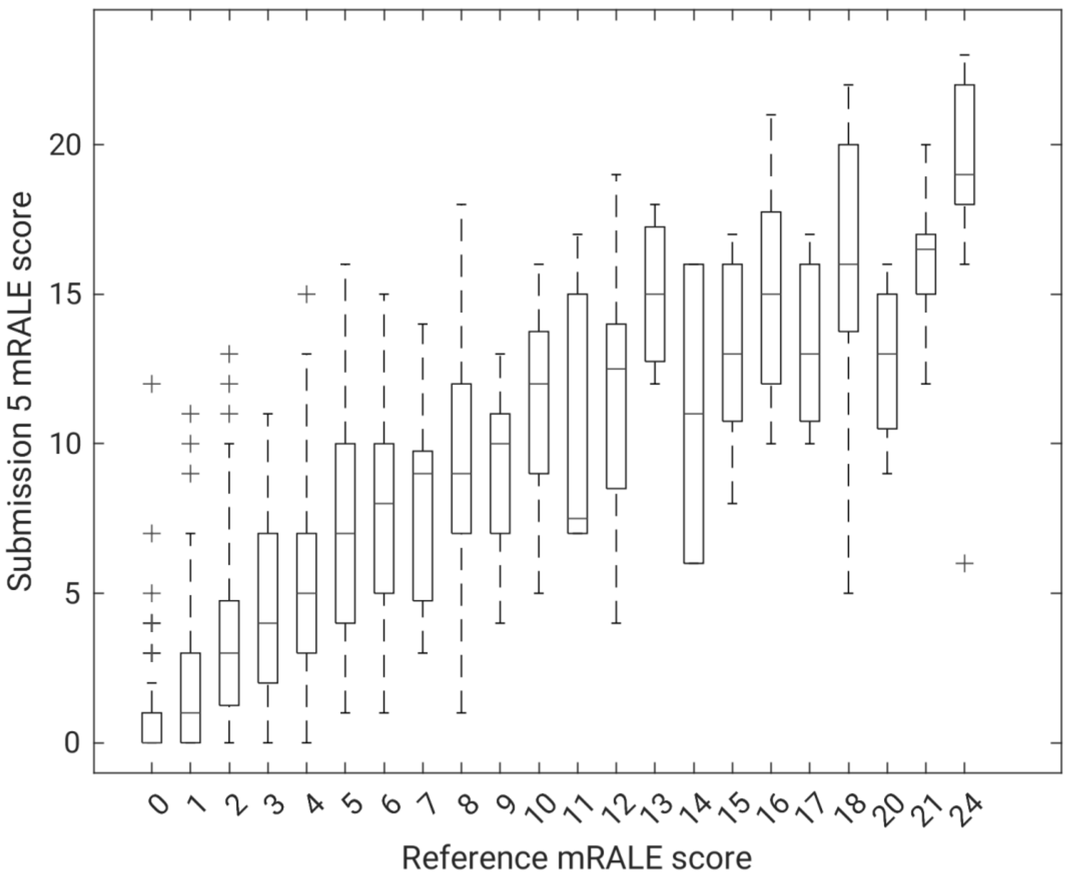

Figure S5. Box-and-whiskers plot for Submission 5 capturing the range of mRALE scores output by the algorithm for the test set cases across the range of test set reference mRALE score.

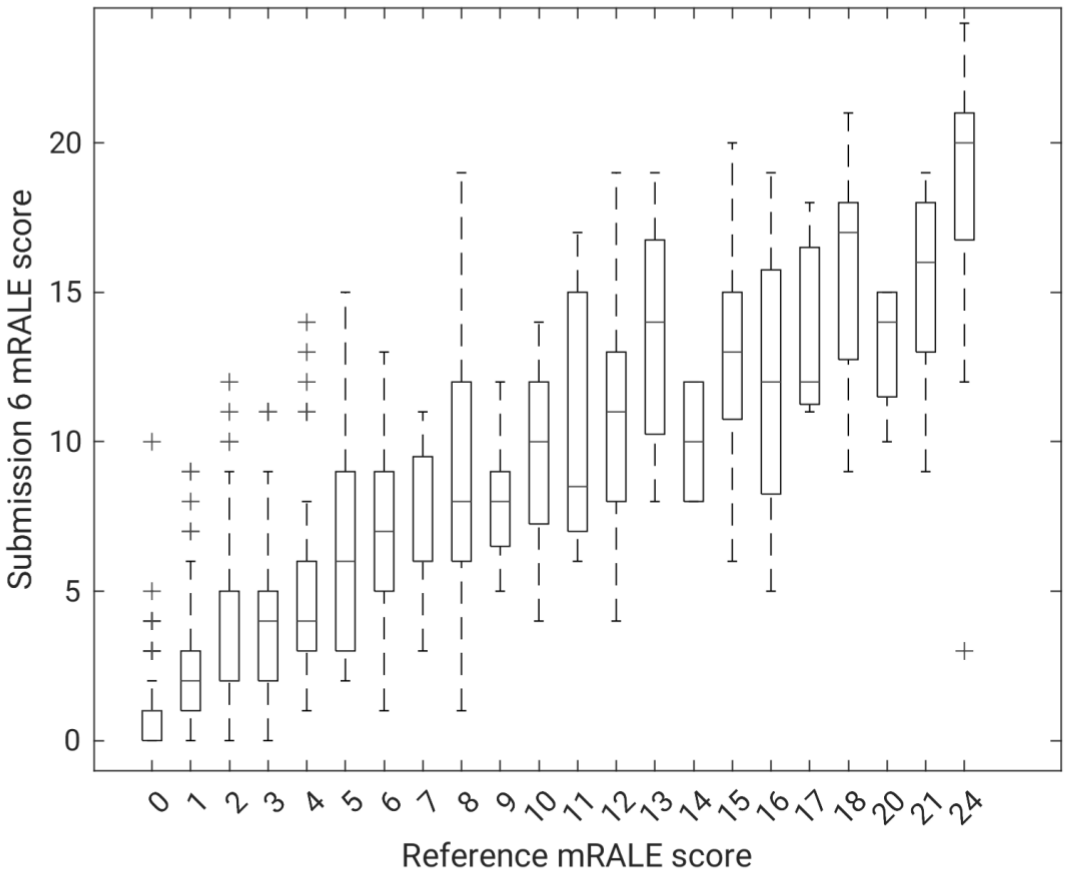

Figure S6. Box-and-whiskers plot for Submission 6 capturing the range of mRALE scores output by the algorithm for the test set cases across the range of test set reference mRALE score.

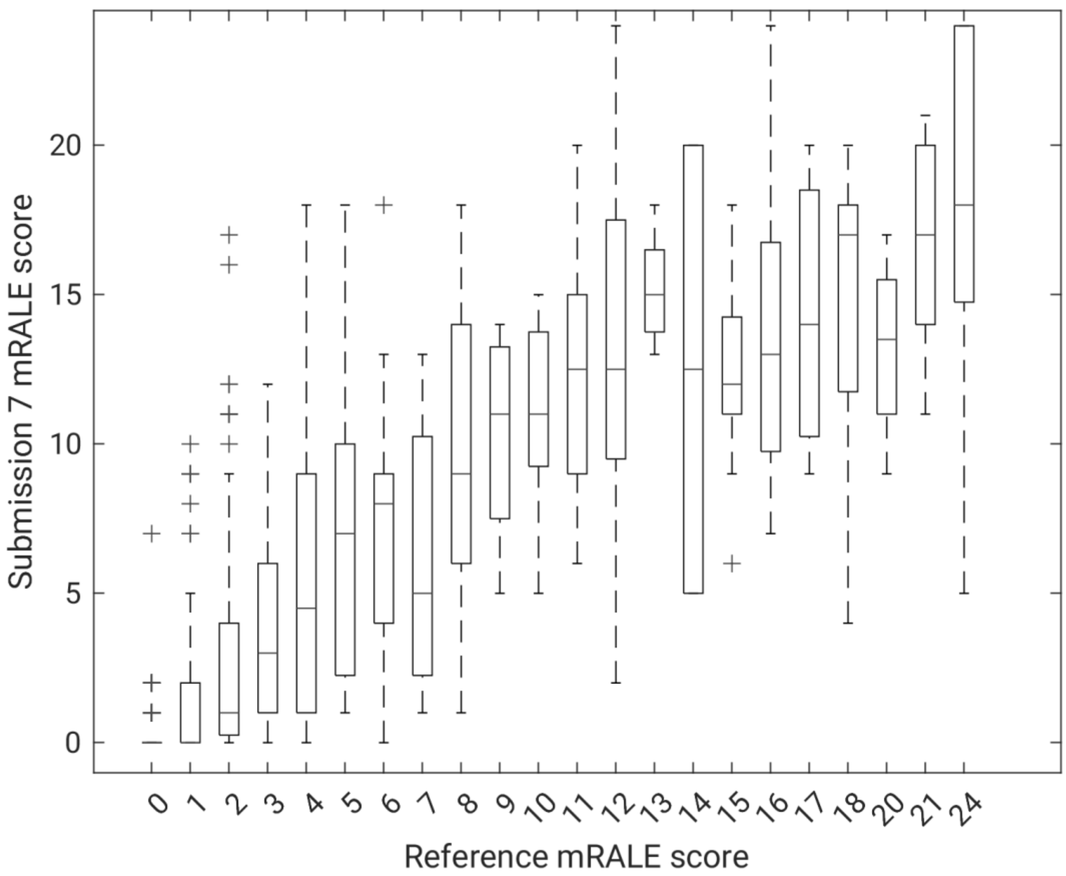

Figure S7. Box-and-whiskers plot for Submission 7 capturing the range of mRALE scores output by the algorithm for the test set cases across the range of test set reference mRALE score.

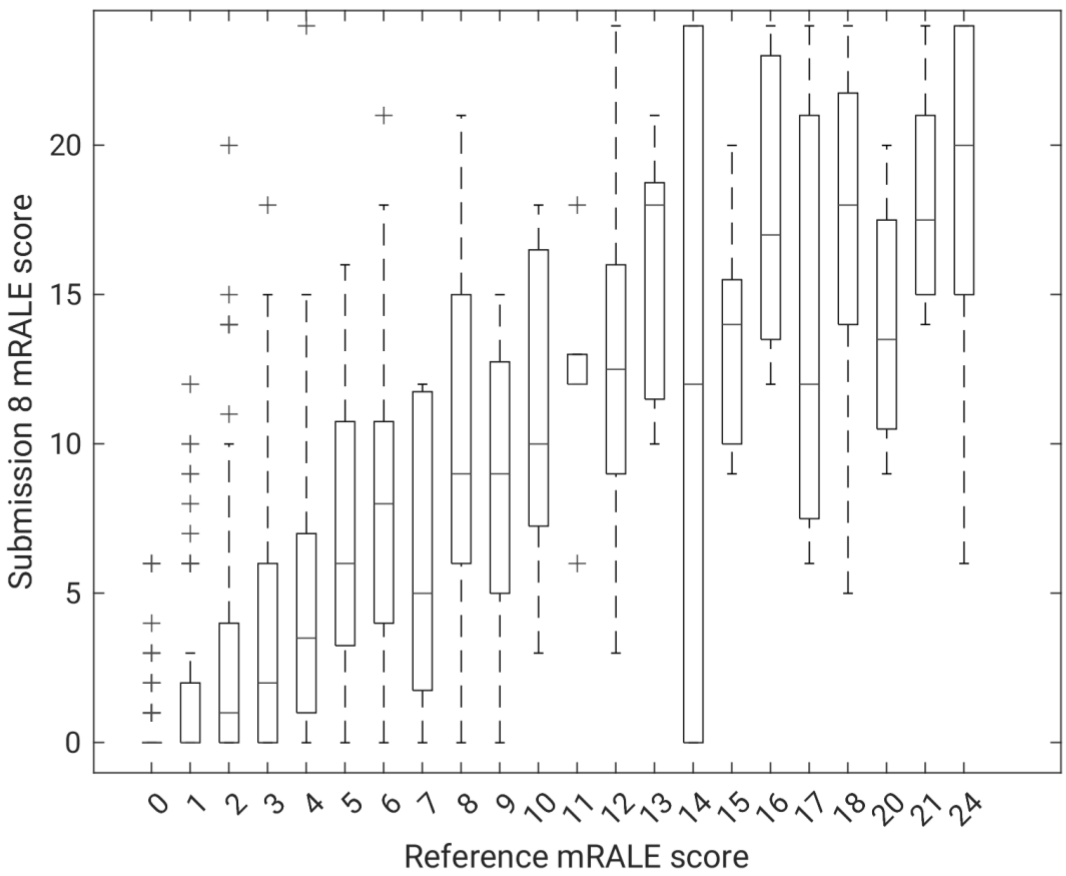

Figure S8. Box-and-whiskers plot for Submission 8 capturing the range of mRALE scores output by the algorithm for the test set cases across the range of test set reference mRALE score.

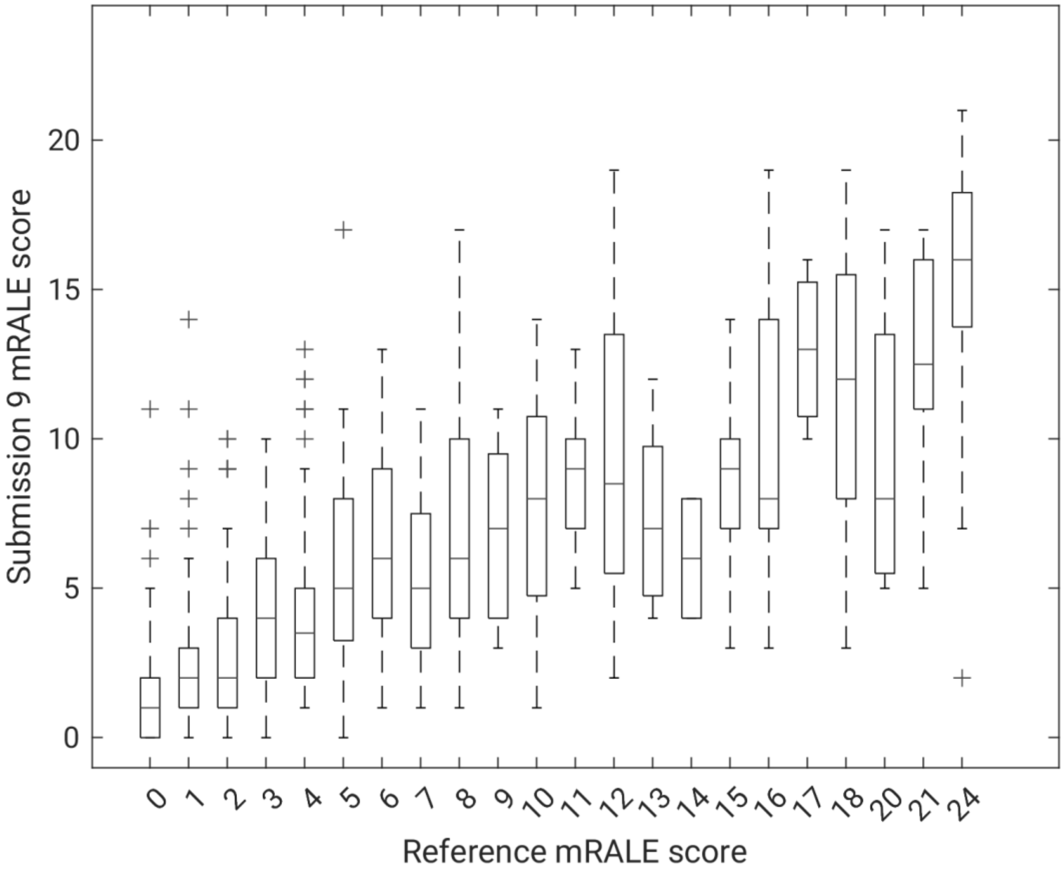

Figure S9. Box-and-whiskers plot for Submission 9 capturing the range of mRALE scores output by the algorithm for the test set cases across the range of test set reference mRALE score.
